# Mini Review: Antimicrobial Control of Chlamydial Infections in Animals: Current Practices and Issues

**DOI:** 10.3389/fmicb.2019.00113

**Published:** 2019-02-04

**Authors:** Sankhya Bommana, Adam Polkinghorne

**Affiliations:** The Animal Research Centre, University of the Sunshine Coast, Sippy Downs, QLD, Australia

**Keywords:** *Chlamydia*, treatment failure, tetracycline resistance, antichlamydials, veterinary medicine, veterinary chlamydiae, antimicrobial treatment

## Abstract

*Chlamydia* are a genus of successful obligate intracellular pathogens spread across humans, wildlife, and domesticated animals. The most common species reported in livestock in this genus are *Chlamydia abortus*, *Chlamydia psittaci*, *Chlamydia suis*, and *Chlamydia pecorum*. Chlamydial infections trigger a series of inflammatory disease-related sequelae including arthritis, conjunctivitis, pneumonia, and abortion. Other bacteria in the phylum Chlamydiae have also been reported in livestock and wildlife but their impact on animal health is less clear. Control of chlamydial infections relies on the use of macrolides, fluoroquinolones, and tetracyclines. Tetracycline resistance (TET^R^) reported for porcine *C. suis* strains in association with the use of tetracycline feed is a potentially significant concern given experimental evidence highlighting that the genetic elements inferring TET^R^ may be horizontally transferred to other chlamydial species. As documented in human *Chlamydia trachomatis* infections, relapse of infections, bacterial shedding post-antibiotic treatment, and disease progression despite chlamydial clearance in animals have also been reported. The identification of novel chlamydiae as well as new animal hosts for previously described chlamydial pathogens should place a renewed emphasis on basic *in vivo* studies to demonstrate the efficacy of existing and new antimicrobial treatment regimes. Building on recent reviews of antimicrobials limited to *C. trachomatis* and *C. suis*, this review will explore the use of antimicrobials, the evidence and factors that influence the treatment failure of chlamydial infections in animals and the future directions in the control of these important veterinary pathogens.

## Introduction

Bacteria within the phylum Chlamydiae are globally significant human and animal pathogens causing asymptomatic infections, as well as acute and chronic diseases in the host. The most well described family in this phylum is the Chlamydiaceae, consisting of 13 taxonomically classified chlamydial species (Sachse et al., 2014) and three *Candidatus* species (Vorimore et al., 2013; Taylor-Brown et al., 2016, 2017; Staub et al., 2018): *C. trachomatis*, *C. muridarum*, *C. suis*, *C. psittaci*, *C. abortus*, *C. caviae*, *C. felis*, *C. pneumoniae*, *C. pecorum*, *C. avium*, *C. gallinacea*, *C. serpentis*, *C. poikilothermis*, *Candidatus C. ibidis*, *Ca. C. corallus*, and *Ca. C. sanzinia*. Outside of the family Chlamydiaceae within the phylum Chlamydiae, significant taxonomic diversity awaits to be discovered with novel families and new species regularly described.

**FIGURE 1:**
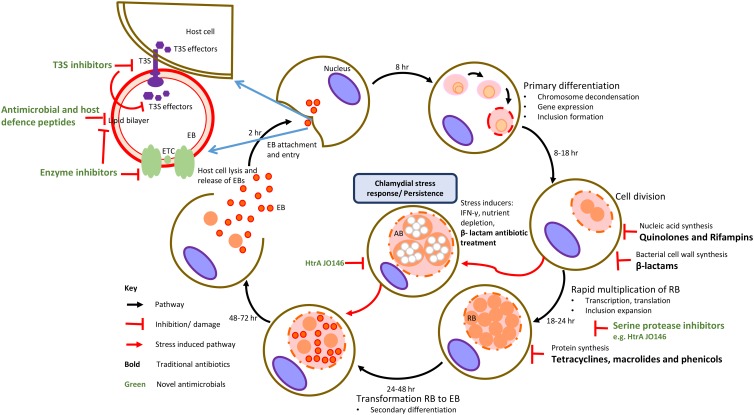
Traditional targets of antimicrobial compounds at various stages of chlamydial developmental cycle. The traditional targets of current class of antibiotics (in black text) are DNA or RNA synthesis, protein synthesis and cell wall synthesis. Novel targets are chlamydial virulence factors, membrane structures and enzymes involved in metabolism with examples of these inhibitors indicated (in green text).

A common feature of these bacteria is a unique and complex intracellular biphasic developmental cycle (Figure 1). The cycle begins when a chlamydial EB attaches to the host cell, is internalized and forms a membrane bound cytoplasmic inclusion. In the inclusion, the EB develops into a non-infectious RB whereby the cell now actively replicates, parasitizing the host cell for metabolites that it acquires through its inclusion. Subsequent populations of RBs mature into infectious EBs that are then released upon host cell lysis to then infect neighboring cells (Abdelrahman and Belland, 2005). During sub-optimal growth conditions, antibiotic treatment or viral co-infection, chlamydial RBs may enter into a non-replicative, non-infective state, yet remaining viable until optimal growth conditions are restored (Bavoil, 2014; Figure 1). Evidence of this chlamydial stress response *in vivo* is rarer with some *in vitro* and *in vivo* evidence of β-lactam-induced persistence reported (Phillips-Campbell et al., 2012; Figure 1).

Chlamydiae are regularly reported in domesticated (Borel et al., 2018) and wild animals (Burnard and Polkinghorne, 2016). In livestock, chlamydial infections of pigs, cattle, sheep, goats, horses and poultry can cause major economic impacts and production losses, worldwide (Borel et al., 2018). *C. suis*, *C. psittaci*, *C. abortus*, and *C. pecorum* are the major livestock pathogens with clinical manifestations ranging from conjunctivitis, arthritis, reproductive disease, and pneumonia posing significant impacts on animal health and economic loss (Borel et al., 2018). Bacteria outside of the genus *Chlamydia* but in the broader phylum Chlamydiae have also been reported in animals (including fish notably) and humans with associations to adverse reproductive outcomes, respiratory infections, and potential zoonosis (Taylor-Brown et al., 2015; Taylor-Brown and Polkinghorne, 2017).

In the near-complete absence of viable chlamydial vaccines for any host, administration of antibiotics and, in particular, the use of tetracyclines, macrolides (inhibitors of protein synthesis), quinolones and rifampins (inhibitors of nucleic acid synthesis) is required for control (Kohlhoff and Hammerschlag, 2015; Figure 1). While the use of these antibiotics is widely accepted, there is growing concern over the emergence of phenotypic antibiotic resistance and treatment failure in the chlamydiae. While most of the attention has focused on treatment failure in humans (Somani et al., 2000; Kong and Hocking, 2015; Kong et al., 2015), the strongest evidence for this is actually in animals where genetically stable TET^R^ and sulfadiazine resistance in *C. suis* strains infecting pigs has been well documented (Sandoz and Rockey, 2010; Borel et al., 2016). Studies of genetically acquired and phenotypic antibiotic resistance patterns in environmental chlamydiae have thus far revealed a similar trend to that of the traditional Chlamydiaceae (Baud and Greub, 2011), although there appear to be exceptions (Vouga et al., 2015).

General information on antimicrobial therapy and its associated complications with therapy failure, genotypic and phenotypic resistance in veterinary chlamydial infections is very limited. To expand on these issues, this review will summarize and discuss the evidence for the use of antimicrobials in the control of veterinary chlamydiae.

## Antimicrobial Treatment of Veterinary Chlamydial Pathogens

Over the last six decades, control of the major veterinary chlamydial pathogens (*C. abortus*, *C. psittaci*, and *C. pecorum*) has centered on the use of tetracycline via TET-supplemented feed. Long acting oxytetracycline or its derivative doxycycline can also be administered orally with the duration and dose varying based on individual farm management practices and the form of tetracycline used for treatment (Ungemach et al., 2006). The mode of action of this antibiotic involves inhibition of chlamydial protein synthesis by binding of the antibiotic to the 30S ribosomal subunit (Figure 1). Additionally, doxycycline also has anti-inflammatory and immunomodulatory properties that result from inhibition of inducible nitric oxide synthase and proinflammatory cytokines (Sykes and Papich, 2014). While the tetracycline class of drugs have been the frontline antichlamydials in the treatment of uncomplicated chlamydial infections, macrolides, phenicols (protein synthesis inhibitors), quinolones, rifampins (nucleic acid synthesis inhibitors), and rarely β-lactams (bacterial cell wall synthesis inhibitors) antibiotic classes have also been successful in treating chlamydial infections (Borel et al., 2016; Figure 1). In the following sections, the use of antibiotics for treating the major veterinary chlamydial pathogens, *C. suis*, *C. abortus*, *C. psittaci*, and *C. pecorum* will be reviewed.

### Treatment of *C. abortus* Infections

*Chlamydia abortus* is the causative agent of EAE and a zoonotic pathogen posing potential threat to pregnant women when in contact with infected ewes (Table 1). Globally, *C. abortus* is a serious cause of economic loss to the sheep production industry (Pospischil et al., 2002; Longbottom and Coulter, 2003). Treatment of early abortion and suspected EAE involves long-acting oxytetracycline (20 mg/kg) during the last month of pregnancy flock-wide (Supplementary Table 1). This administration has been shown to reduce the severity of *C. abortus* infections, pathological damage and eventually to increase the chances of live birth (Aitken et al., 1982; Greig et al., 1982). Usually a single dose is recommended to avoid emergence of TET resistance, however, fortnightly routine administration (oral tetracycline type product included in the feed at 400–500 mg/hd/day) until lambing seems to further suppress chlamydial shedding, which is crucial to prevent excretion of *C. abortus* at birth as well as on-farm spread of the infection (Rodolakis et al., 1980; Supplementary Table 1). Prophylactic use of tetracycline could potentially lead to emergence of acquired TET resistance, moreover, the use of therapy does not guarantee eradication of *C. abortus* infection with a small percentage of the pregnant flock still producing stillborn and weak born lambs whilst potentially carrying *C. abortus* post-treatment (Essig and Longbottom, 2015; Rodolakis and Laroucau, 2015; Table 1). To date tetracycline-resistant strains in *C. abortus* have not been isolated yet, however, relapse of infection or presence of *C. abortus* shedding post-treatment is suggestive of treatment failure due to the establishment of antibiotic protected reservoirs. Simultaneous detection of *C. suis* and *C. abortus* in semen of boars and conjunctiva of sows in pig production has been reported (Schautteet et al., 2010), further highlighting the potential risk of the spread of TET^R^ resistance to other animal chlamydiae if significant selective pressure is maintained (Suchland et al., 2009). Despite being a major veterinary pathogen of zoonotic importance, there appears to be a lack of *in vitro* and *in vivo* models/studies investigating the role of antibiotics in the treatment of *C. abortus* infections in humans and animals.

**Table 1:** Members of the order Chlamydiales and their animal host pathogenicity, antibiotic susceptibility, treatment and resistance.

Species	Pathogenicity in animals	Antibiotic susceptibility	Treatment	Resistance/treatment failure/*in vitro* evidence of antibiotic-induced persistence	Reference
*Chlamydia suis*^∗^	Respiratory disease, diarrhea, conjunctivitis, and reproductive disorders in pigs	Rifaximin, levofloxacin, and doxycycline	Aminoglycoside; β-lactams; fluoroquinolone; or tetracycline. Pro-/metaphylactic herd treatment: amoxicillin; chlortetracycline; MDT – chlortetracycline, sulfadimidine, tylosin; or MDT – trimethoprim, sulfadimidine, sulfathiazole.	Tetracyclineˆ and sulfadiazineˆ	Hoffmann et al., 2015
*Chlamydia abortus*^∗^	Ovine enzootic abortion	Tetracycline	Tetracycline, oxytetracycline, erythromycin, and clarithromycin.	–	Aitken et al., 1982
*Chlamydia psittaci*^∗^	Respiratory, joint, and reproductive disease in poultry, cattle, and horses	Doxycycline and enrofloxacin	Tetracycline, doxycycline, and rifampicin.	β-lactams ^$^, tetracycline^#^, and rifampicin^#^	Butaye et al., 1998; Goellner et al., 2006
*Chlamydia pecorum*	Ruminants: joint and ocular disease Koalas: ocular, urogenital, and reproductive disease	Tetracycline (ruminants), chloramphenicol, and florfenicol (koalas)	Tetracycline, chloramphenicol, and florfenicol.	β-lactams ^$^	Pudjiatmoko et al., 1998; Black et al., 2015; Leonard et al., 2017
*Parachlamydia acanthamoebae^∗^*	Miscarriage and pneumonia in bovines	Macrolides, tetracyclines, and rifampin	Azithromycin, clarithromycin, and/or doxycycline.	Quinolonesˆ, amoxicillinˆ, ceftriaxoneˆ, and imipenemˆ	Greub, 2009; Vouga et al., 2015
Simkaniaceae*^∗^*	Granulomatous lesions in reptiles	Macrolides, clindamycin, cyclines, rifampin, and quinolones	azithromycin, clarithromycin, and/or doxycycline.	β-lactams ˆ, fosfomycinˆ, and vancomycinˆ	Friedman et al., 2003; Vouga et al., 2017
*Waddlia chondrophila^∗^*	Miscarriage and pneumonia in cattle	Doxycycline and azithromycin	Azithromycin, clarithromycin, and/or doxycycline^@^.	β-lactams ˆ and fluoroquinolonesˆ	Goy and Greub, 2009
Environmental CLOs	Epitheliocystis in fish	Tetracycline	Oxytetracycline.	Enrofloxacin^#^	Goodwin et al., 2005; Polkinghorne et al., 2010

*^∗^Zoonotic and/or pathogenic in humans. ^∧^Antibiotic resistance. ^#^Antibiotic treatment failure. ^$^In vitro evidence of antibiotic-induced chlamydial stress response. ^@^Empirical antimicrobial treatment in the absence of in vivo efficacy data.*

### Treatment of *C. psittaci* Infections

*Chlamydia psittaci* is an avian pathogen capable of causing systemic wasting disease in wild birds and production species such as chickens and ducks (Knittler and Sachse, 2015). Infection spill-over to other hosts is also a concern with *C. psittaci* recognized as a serious zoonotic agent of atypical pneumonia in humans (Stewardson and Grayson, 2010; Knittler and Sachse, 2015) with evidence growing for spill-over of infections and disease to other mammalian hosts as well (Van Loo et al., 2014; Jenkins et al., 2018). Human cases of psittacosis are effectively treated using orally administered doxycycline and tetracycline hydrochloride for a period of 10–14 days (Beeckman and Vanrompay, 2009; Senn et al., 2005). In patients for whom tetracycline is contra-indicated, i.e., in pregnant woman and children under the age of 8 years treatment with azithromycin and erythromycin at a dose of 250–500 mg PO qd for 7 days has proven to be the best alternative (Senn et al., 2005; Beeckman and Vanrompay, 2009). This is probably the main reason for the general decline in psittacosis cases worldwide, particularly those with fatal outcome, in the past decades. However, use of quinolones to treat chlamydia infections in humans has resulted in reports of treatment failure (Beeckman and Vanrompay, 2009).

The antibiotics of choice in veterinary medicine for the treatment of *C. psittaci* infections are doxycycline or other tetracyclines and the fluoroquinolone enrofloxacin administered orally (feed/drinking water) or parenterally (intramuscular or subcutaneous routes) (Flammer, 1989; Butaye et al., 1998; Supplementary Table 1). In terms of persistent infections, studies in bovine respiratory models have shown that treatment with tetracycline or rifampicin revealed evidence of clinical recovery of respiratory symptoms although re-isolation of the organism was still possible in some animals with no significant reduction in chlamydial shedding 14-days post-treatment of antibiotics (Prohl et al., 2015a,b; Table 1). *In vitro* studies also suggest that the development of drug-resistant *C. psittaci* strains is possible (Binet and Maurelli, 2005) and that *C. psittaci* is also capable of entering a persistent state upon treatment with penicillin G conceivably playing a role in the development of chronic infections, as well as in failure of antibiotic therapy and immunoprophylaxis (Goellner et al., 2006; Table 1). Although, TET^R^ seems to be a problem only in *C. suis*, lack of antimicrobial resistance screening in routine diagnostic testing from *C. psittaci* field isolates in poultry and cattle impairs the assessment of the actual situation. While treatment with doxycycline and/or azithromycin seems to be efficacious for *C. psittaci* infections in birds, the widespread use of tetracycline in feed and/drinking water and long periods of treatment (21–25 days) in the poultry and bird industry (Guzman et al., 2010; Krautwald-Junghanns et al., 2013) can also lead to an accumulation of sub-therapeutic drug plasma concentrations (Tell et al., 2003), supporting the emergence of drug-resistant *C. psittaci* strains (Supplementary Table 1). Subclinical, persistent and chronic disease and infection relapse post-treatment is also plausible suggesting that there is need for pre- and post-antimicrobial treatment surveillance of *C. psittaci* infections in animals.

### Treatment of *C. suis* Infections

Antibiotic therapy and the associated resistance reported for *C. suis* have been thoroughly reviewed recently (Borel et al., 2016). Briefly, *C. suis* is an endemic GIT pathogen of pigs. While a range of pathologies have been reported in association with *C. suis* infection (respiratory disease, diarrhea, conjunctivitis and reproductive disorders), the high rates of GIT positivity for this pathogen are commonly reported in the absence of disease (Schautteet and Vanrompay, 2011). Due to the endemic nature of *C. suis* in most pig production facilities, infections are rarely treated by antibiotics such as oxytetracycline. Quinolones (enrofloxacin) or macrolides (erythromycin) can be administered, in case of an infection with a TET^R^ *C. suis* strain (Schautteet and Vanrompay, 2011; Table 1). However, due to emergence of TET^R^ in *C. suis*, alternative treatment strategies such as the short-term treatment of *C. suis* infections with enrofloxacin and tiamulin was unsuccessful resulting in recurrence of *C. suis* infections in pigs (Reinhold et al., 2011a). The TET^R^ feature of this bacterium, namely that it is the first and only species of intracellular bacteria known to have genetically acquired antibiotic resistance (Sandoz and Rockey, 2010) is of significant interest. The basis of this stable TET^R^ phenotype is the presence of a Tet-island in the genome of *C. suis*, consisting of tetC gene encoding a TET efflux pump, TET repressor gene (tetR) (Dugan et al., 2004). These loci share high nucleotide sequence identity with several other Gram-negative bacterial-resistance plasmids, one of them being the fish bacteria *Aeromonas salmonicida* mobilizable plasmid pRAS3.2 (Dugan et al., 2004). Expanded studies of this Tet-island found that even in very distinct *C. suis* evolutionary lineages, this Tet island is present in the same genomic location adjacent to an rRNA operon (Seth-Smith et al., 2017). Based on studies at the herd-level, antibiotic treatment appears to promote the emergence of TET^R^ and further spread of this resistance cassette among Tet-sensitive *C. suis* strains (Hoffmann et al., 2015; Wanninger et al., 2016).

It should be noted that acquisition of Tet Island is associated with mobile genetic elements, raising concerns over the potential spread and distribution of these elements across diverse set of bacteria, particularly into *C. trachomatis*, the most closely related currently described chlamydial species to *C. suis*. This potential risk has been confirmed experimentally with studies showing that *C. suis* can confer TET^R^ to *C. trachomatis in vitro* (Suchland et al., 2009). Further highlighting this risk, *C. suis* has also been documented in ocular infections in humans with trachoma (Dean et al., 2013) and in workers in a pig production facility (De Puysseleyr et al., 2014). While most of the current risk of *C. suis* TET^R^ resistance is confined to pigs, *C. suis* has also been detected in other animals including livestock, horses, cats, poultry (Teankum et al., 2006; Polkinghorne et al., 2009; Pantchev et al., 2010; Szymanska-Czerwinska et al., 2013; Guo et al., 2016) and wildlife (e.g., frogs) (Blumer et al., 2007).

### Treatment of *C. pecorum* Infections

There is limited information on the efficacy of antibiotics against *C. pecorum*, with a single *in vitro* study suggesting that livestock isolates of this pathogen are susceptible to macrolides, tetracyclines and quinolones with potential recovery upon removal of the antibiotic not evaluated to further understand chlamydial latency (Pudjiatmoko et al., 1998; Table 1). In practice, treatment of *C. pecorum*-infected animals displaying evidence of arthritis, sporadic bovine encephalitis and conjunctivitis involves the use of intramuscular injections of long-acting oxytetracycline (300 mg/mL at a dose rate of 1 mL per 10 kg bodyweight) once a week, twice (Walker et al., 2016; Supplementary Table 1). Potential issues of chlamydial latency rather than infection clearance (Mårdh and Löwing, 1990; Smith, 2002) have been reported in association with this treatment with detectable chlamydial DNA loads as high as pre-treatment levels, three to 6 weeks post-treatment reported in some studies (Parkinson et al., 2010; Reinhold et al., 2011b; Walker, 2013; Table 1). *In vitro* data also suggests that penicillin G induces the chlamydial stress response (persistence) and is not bactericidal for this chlamydial species (Leonard et al., 2017; Table 1). This is of particular concern in livestock production industry where it is likely that some animals with endemic, asymptomatic *C. pecorum* infection are treated with both veterinary-approved and off-label antibiotics for other infections and purposes.

The treatment of *C. pecorum* infections is also of relevance to the veterinary treatment of the iconic Australian marsupial, the koala. Koalas infected by *C. pecorum* can develop ocular and urogenital tract disease that may lead to animals being admitted into wildlife hospitals for veterinary treatment (Polkinghorne et al., 2013). Treatment of clinical and subclinical koala chlamydiosis most commonly involves the administration of chloramphenicol due to its perceived safety and anecdotal effectiveness, despite a lack of information on therapeutic efficacy or pharmacokinetics in this marsupial host (Black et al., 2015; Table 1). Chloramphenicols are preferred over the efficacious first-line antichlamydials, azithromycin, or tetracyclines, as use of the latter antibiotics have been associated with gastrointestinal dysbiosis and emaciation in koalas (Osawa and Carrick, 1990). Summary of treatment regimens and associated complications of ocular, urogenital and reproductive tract disease in koalas have been reviewed in detail and can be found elsewhere (Vogelnest and Portas, 2018; Supplementary Table 1).

### Treatment of *Chlamydia*-Related Bacteria (CRBs)

The discovery of new family level lineages in the order Chlamydiales such as the Parachlamydiaceae, Simkaniaceae, Criblamydiaceae, and Waddliaceae has prompted investigations into the pathogenic potential of this bacteria. Thus far, a range of studies have suggested that these bacteria may be linked to adverse pregnancy outcomes and respiratory disorders in humans and animals, with animal contact as a potential risk factor for higher prevalence (Taylor-Brown et al., 2015; Ammerdorffer et al., 2017; Borel et al., 2018; Table 1). While the pathogenic potential of these chlamydiae is yet to be fully defined, more recently described chlamydiae spread across several family-level taxonomic groups are well recognized causes of the gill disease of fish, epitheliocystis (Blandford et al., 2018).

There are very limited studies reported so far on antibiotic treatment regimens for CRBs in humans and animals with most of the knowledge of antibiotic efficacy and/or phenotypic resistance based on *in vitro* studies (Friedman et al., 2003; Goy and Greub, 2009; Greub, 2009; Vouga et al., 2015, 2017). These *in vitro* studies have revealed that most CRBs are resistant to quinolones and β-lactams with *Parachlamydia* and *Neochlamydia* spp. also demonstrating phenotypic resistance to amoxicillin, ceftriaxone and imipenem (MIC ≥32 μg/ml) (Vouga et al., 2015; Table 1). In the absence of data from animal models and from case reports, azithromycin, clarithromycin and/or doxycycline might be used therapeutically in case of *Parachlamydia acanthamoebae* infections (Greub, 2009). For *Simkania*, a single case study reported that simkania-associated pneumonia was successfully treated with a regimen of erythromycin (Lieberman et al., 1997). Oxytetracyclines have been found to be effective in treating epitheliocystis infections in several fish species, usually, mixed in the feed at a dose of 50 mg/kg/d for 3–5 consecutive days (Goodwin et al., 2005; Chang et al., 2016; Supplementary Table 1). Enrofloxacin failed to treat a leopard shark with epitheliocystis (Polkinghorne et al., 2010). Apart from the use of antibiotics in aquaculture, several alternative strategies have been used for treating epitheliocystis such as sterilization of rearing water using ultraviolet light (Miyaki et al., 1998), chemical treatments such as formalin, salt, benzalkonium chloride, potassium permanganate, and water exchange (Somridhivej et al., 2009; Blandford et al., 2018).

## Future Directions and Concluding Remarks

While there is extensive clinical evidence supporting the use of antibiotics for the treatment of the most common chlamydial infections, this review has highlighted that, for most veterinary chlamydiae, comprehensive *in vivo* studies of the efficacy of these antibiotics have not been performed till date. This is obviously concerning given the growing body of evidence to suggest the potential for chlamydial antimicrobial resistance and treatment failure and the patterns and underlying causes of antibiotic resistance, treatment failure and relapse of infection post antibiotic treatment.

This issue becomes even more pressing when considering the continuing new information emerging about the host range of previously described chlamydiae as well as the range of novel chlamydiae being detected in animals (Taylor-Brown and Polkinghorne, 2017). In terms of the former, there is growing evidence that the ‘host barriers’ previously defined for veterinary chlamydiae are looser than first thought, with evidence that important chlamydial pathogens such as *C. psittaci* can infect a diverse range of animal hosts (Knittler et al., 2014) while others such as *C. caviae* are a more serious zoonotic risk than previously thought (Ramakers et al., 2017). Antimicrobial efficacy studies are lacking to inform treatment options in these new host species. In the absence of such information for novel chlamydiae, clinicians have no choice but to use treatment regimens used for existing chlamydiae. For example, the reported treatment for the newly emerging and apparently widespread chlamydial agent, *C. gallinacea*, involves tetracyclines or macrolides based on the treatment regimen for *C. psittaci* infections in poultry (Brown et al., 2016). Recent studies describing antibiotic sensitivity to tetracycline and moxifloxacin and phenotypic resistance to azithromycin in the newly described chlamydial species infecting snakes, *C. serpentis* and *C. poikilothermis* (Staub et al., 2018), demonstrate the potential for considerable variability in the antibiotic resistance profile of bacteria in the genus *Chlamydia*. As new chlamydial species continue to emerge in animals, the demonstration or prediction of antibiotic resistance to inform clinical treatment of these infections will become increasingly important.

In terms of the factors that may influence the success of antimicrobial control in animals, the clearance of GIT infections is still a major concern for existing antimicrobials and novel antichlamydials under development (Yeruva et al., 2013; Zhang et al., 2015). The intestinal site appears to be a natural habitat for infection of chlamydiae infecting mammalian and avian hosts, wherein studies have reported long-term GIT infections with continual shedding of the pathogen in the feces (Meyer and Eddie, 1933; York and Baker, 1951; Yang et al., 2014). This is particularly important because GIT infections associated with fecal shedding in flocks and herds appear to be the precursor to abortion, encephalitis, polyarthritis, conjunctivitis, and pneumonia in ruminants (Campos-Hernández et al., 2014; Hoffmann et al., 2015; Walker et al., 2016; Bommana et al., 2018). Ruminal and small animal models also suggest that neither the host immune system nor the use of antimicrobials is successful in clearing chlamydiae from the gut (Yeruva et al., 2013; Rank and Yeruva, 2014), due to the establishment of an antibiotic-protected reservoir in the GIT and down regulatory mechanisms further inhibiting the adaptive immune response from resolving GIT infections (Igietseme et al., 2001). Future studies to demonstrate the *in vivo* efficacy of existing and novel anti-chlamydial agents will need to account for GIT infection reservoirs if chlamydial “cure,” rather than clearance of symptoms, is the goal of such treatment.

In terms of acquired antibiotic resistance, co-infections of *C. suis* with *C. trachomatis* in humans and veterinary chlamydial species in animals poses the potential threat for horizontal transfer of TET^R^ in these chlamydial species with tetracycline sub-therapeutic treatment/dosing potentially inducing selective pressure for emergence of TET^R^. The emergence of antibiotic resistance should be the driver for development and application of new antichlamydials in veterinary medicine. Efforts for these are already underway, including in animals (Lawrence et al., 2016), exploiting a range of strategies to target *Chlamydia*-specific cell structures and/or known virulence factors (Ur-Rehman et al., 2012; Marti et al., 2014; Koroleva et al., 2015; Rahn et al., 2016; Donati et al., 2017; Papa et al., 2017; Figure 1). Regardless of years of research into chlamydia control through immunoprophylaxis there are almost no viable chlamydial vaccines to date. In case of *C. abortus*, there is a live vaccine used in Europe and elsewhere that has been greatly beneficial in reducing the use of antimicrobials and emergence of resistance in sheep production, however, use of this live vaccine has been linked to the more recent OEA outbreaks and vaccine breakdown (Wheelhouse et al., 2010; Longbottom et al., 2018). Such efforts will be vital to meeting the demands to continue to control chlamydial infections in animals successfully in the 21st century.

## Author Contributions

SB and AP conceptualized and wrote the manuscript.

## Conflict of Interest Statement

The authors declare that the research was conducted in the absence of any commercial or financial relationships that could be construed as a potential conflict of interest.

## Abbreviations

EAE: enzootic abortion of ewes
EB: elementary body
CRBs: *Chlamydia*-related bacteria
GIT: gastrointestinal tract
MIC: minimum inhibitory concentration
RB: reticulate body
TET^R^: tetracycline resistance.
