# Supplementary material for: Mini Review: Antimicrobial Control of Chlamydial Infections in Animals: Current Practices and Issues
Source: Front Microbiol. 2019 Feb 4;10:113. doi: 10.3389/fmicb.2019.00113 (PMC6369208; doi:10.3389/fmicb.2019.00113)
Supplement: Supplementary file 1 [file Table_1.DOCX]

Supplementary Material

**Antimicrobial Control of Chlamydial Infections in Animals: Current Practices and Issues**

**Sankhya Bommana^*^ and Adam Polkinghorne**

***Correspondence:**

Sankhya Bommana

Sankhya.bommana@research.usc.edu.au

Supplementary Table 1. Antibiotic treatment regimen, dose and administration in hosts with chlamydial infections.

| **Host** | **Species** | **Antibiotic treatment** | **Dose** | **Route of administration** | **Reference** |
| --- | --- | --- | --- | --- | --- |
| Sheep | *C. abortus* | Long-acting oxytetracycline | 20mg/kg; two injections at two weeks interval during the last month of pregnancy | Intradermal injection | Rodolakis et al., 2015 |
|  | *C. abortus* | Oral tetracycline | 400-500mg/herd/day fortnightly routine administration until lambing | Oral feed | Rodolakis et al., 1980 |
|  | *C. pecorum* | Long-acting oxytetracycline | 300 mg/mL at a dose rate of 1 mL per 10 kg bodyweight | Intramuscular injection | Walker et al., 2016 |
| Pigs | *C. suis* | Enrofloxacin | 4 mg/kg for 5 days/ 2.5mg/kg for 3 days followed by 100mg/ml tiamulin | Intramuscular injection | Reinhold et al., 2011 |
|  | *C. suis* | Combination of trimethoprime, sulfadimidine and sulfathiazole (TSS) | NA | Prophylactic oral antibiotic treatment | Hoffmann et al., 2015 |
|  | *C. suis* | Combination of chlortetracycline, tylosin and sulfadimidin | NA | Prophylactic oral antibiotic treatment | Hoffmann et al., 2015 |
|  | *C. suis* | Amoxicillin | NA | Therapeutic treatment | Hoffmann et al., 2015 |
| Cattle | *C. pecorum* | Long-acting oxytetracycline/  thiamine and/or fluoroquinolones | NA | Intramuscular injection,  Oral administration through milk in calves | Hunt et al., 2016 |
|  | *C. abortus* | Long-acting oxytetracycline | NA | Intramuscular injection | Walker et al., 2016 |
| Cattle | *C. psittaci* | Doxycycline | 10 mg kg/day twice daily for 14 days | Oral administration | Prohl et al., 2015 |
|  | *Parachlamydia acanthamoebae* | Azithromycin, clarithromycin and/or doxycycline | NA | NA | Greub et al., 2009 |
|  | *Waddlia chondrophila* | Doxycycline and azithromycin | NA | NA | Goy and Greub, 2009 |
| Koalas | *C. pecorum* | Chloramphenicol | 60 mg/kg, single dose per day for 14-28 days | Subcutaneous injection | Vogelnest and Portas, 2018 |
| Birds | *Chlamydia psittaci* | Chlortetracycline, doxycycline or enrofloxacin | 500-5000ppm according to the species and food in case of Chlortetracycline,  1000 mg/kg in case of DOX and 250-1000 ppm in case of enrofloxacin.  Duration of treatment varies. | Oral administration through feed or water and/or Intramuscular injection | Rodolakis et al., 2010; Gerlach, 1999 |
| Fish | Environmental CLOs | Oxytetracycline | 50 mg/kg/d for 3-5 consecutive days | Oral feed | Goodwin et al., 2005; Chang et al., 2016 |
